# Endogenous endophthalmitis caused by group B streptococcus; case reports and review of 35 reported cases

**DOI:** 10.1186/s12886-020-01378-0

**Published:** 2020-03-31

**Authors:** Masaaki Yoshida, Shunji Yokokura, Takashi Nishida, Kiyofumi Mochizuki, Takashi Suzuki, Kazuichi Maruyama, Takaaki Otomo, Koji M. Nishiguchi, Hiroshi Kunikata, Toru Nakazawa

**Affiliations:** 1 Department of Ophthalmology, Tohoku University Graduate School of Medicine, 1-1, Seiryo-machi, Aoba-ku, Sendai, Miyagi 980-8574 Japan; 2 Department of Ophthalmology, Gifu University Graduate School of Medicine, Gifu, Japan; 3 Department of Ophthalmology, School of Medicine, Toho University, Tokyo, Japan; 4 Department of Advanced Ophthalmic Medicine, Tohoku University Graduate School of Medicine, Sendai, Japan; 5 Department of Retinal Disease Control, Tohoku University Graduate School of Medicine, Sendai, Japan; 6 Department of Ophthalmic Imaging and Information Analytics, Tohoku University Graduate School of Medicine, Sendai, Japan

**Keywords:** Endogenous bacterial endophthalmitis, Group B streptococcus (GBS, *Streptococcus agalactiae*), Endocarditis, Diabetes mellitus, Quinolone-resistant GBS

## Abstract

**Background:**

Group B streptococcus (GBS), a gram-positive coccus that occasionally causes neonatal sepsis or invasive infection in the elderly, has been considered a rare cause of endogenous bacterial endophthalmitis (EBE). However, the number of invasive GBS infections is increasing, particularly in elderly patients with underlying conditions such as diabetes mellitus (DM), cardiovascular disease and cancer. We report 6 cases of EBE caused by GBS and review the literature.

**Methods:**

Retrospective case series and literature review.

**Results:**

In the current case series, 6 eyes of 6 patients developed EBE caused by GBS. The average age was 73.5 years. The focus of infection included the urinary tract, cellulitis, arthritis, peritonitis, catheter-associated infection and endocarditis. Four patients had DM. While all 6 strains were sensitive to β-lactams (penicillins and cephems), 4 strains were resistant to levofloxacin (no data for 1 isolate). Each case was treated with the systemic antibiotic to which the individual strain was sensitive. All cases showed poor visual acuity at presentation (decimal visual acuity: less than 0.03). Vitrectomy with intravitreal antibiotics injection was performed in 4 cases. Visual acuity recovered in 4 cases and did not recover in 2 cases, even after vitrectomy. The literature review of 53 eyes of 41 patients revealed that 60% of eyes finally lost all vision, and death occurred in 2 cases. Initial visual acuity of less than counting fingers was associated with a final outcome of lost vision. Of 41 patients, 13 (32%) had DM as an underlying medical condition. The most common extra-ocular infection focus was endocarditis (37%).

**Conclusions:**

DM is common in patients with EBE caused by GBS. While the 4 cases in the current report had a relatively good visual acuity outcome, despite poor initial visual acuity, the literature review indicated that EBE caused by GBS is generally a severe condition with a poor prognosis. The current study also indicates the importance of considering the possibility of endocarditis on encountering EBE caused by GBS.

## Background

Endogenous bacterial endophthalmitis (EBE), a complication of systemic blood-stream infection, is a rare but dangerous threat to vision. Studies from different geographical regions have obtained different results on causative organisms in EBE: East Asian reports found that gram-negative organisms, especially *Klebsiella pneumoniae,* were the leading cause of EBE, while Western reports found that gram-positive organisms such as *Staphylococcus aureus* and *Streptococcus pneumonia* were more frequent [1–4]. Group B streptococcus (GBS; *Streptococcus agalactiae*), a gram-positive coccus that occasionally causes neonatal sepsis or invasive infection in the elderly, has been considered a rare cause of EBE, especially in East Asia. However, the number of invasive GBS infections is increasing, particularly in elderly patients with underlying conditions such as diabetes mellitus (DM), cardiovascular disease and cancer [5–8]. In the last 15 years, our affiliated institutions (Tohoku University Hospital and Gifu University Hospital), located in Japan, have seen at least 6 cases of EBE caused by GBS in elderly patients. Moreover, an online search revealed 10 more cases in Japan [9–18]. Thus, at least 35 cases of EBE caused by GBS in adults have been reported in English or Japanese [2, 9–33]. Although a variety of microorganisms cause EBE, we consider that GBS is one of the most important. Here, we examine the clinical characteristics of EBE caused by GBS, describe 6 cases we observed, and review relevant recent literature, including a comparison of East Asian and Western cases.

## Methods

### Patients

We retrospectively reviewed the records of 6 eyes of 6 patients with EBE caused by GBS, all of whom were observed at Tohoku University Hospital or Gifu University Hospital between December 2003 and September 2016. The diagnosis of EBE caused by GBS was based on positive results from culture testing of blood or ocular samples (either the aqueous humor or vitreous body) obtained from patients with iritis and vitritis during ophthalmic examination. Patients with a potential exogenous cause of infection, such as trauma, recent ocular surgery or corneal ulcer, were excluded. Clinical histories, including underlying medical conditions, initial symptoms, culture testing, infection foci, treatment and final visual acuity outcome were examined. Database searches were performed with Medline for articles in English and Google Scholar for articles in Japanese for the period ending in June 2019. Each search query included the terms “Group B streptococcus”, “*Streptococcus agalactiae*” and “endogenous endophthalmitis”. In Google Scholar, these Japanese equivalents for these terms were used. These searches identified 47 eyes of 35 patients with EBE caused by GBS (Medline: 36 eyes of 29 patients in 18 English articles, Google Scholar: 11 eyes of 6 patients in 6 Japanese articles). Thus, combined with the 6 eyes of 6 patients observed directly, this study retrospectively reviewed the records of 53 eyes of 41 patients.

### Microbiological studies

At Tohoku University Hospital, isolated colonies on 5% sheep blood agar plates were evaluated with matrix-assisted laser desorption/ionization-time of flight mass spectrometry (MALDI-TOF MS) using the VITEK MS ver. 3.0 (bioMérieux, Marcy L’Étoile, France); this revealed that the colonies comprised *Streptococcus agalactiae*. At Gifu University Hospital, identification of GBS was performed with Gram staining, a catalase reaction, examination of colony morphology, hemolysis with a blood agar plate, and an examination for the Lancefield group B antigen. Susceptibility testing for antibiotics was performed with the microdilution method at both participating institutions. The MicroScan WalkAway 96 plus System (Beckman Coulter Inc., Brea, CA, USA) with a MICrofast7J panel was used at Tohoku University Hospital and the RAISUS system (Nissui Pharmaceutical Co., Ltd., Tokyo, Japan) with an NKMP1 plate was used at Gifu University Hospital. All results were interpreted according to the Clinical and Laboratory Standards Institute standard method.

### Statistical analysis

Fisher’s exact test was used to compare binary data. *P* values < 0.05 were considered statistically significant.

## Results

### Observational case series

A clinical summary of the 6 cases observed at our clinics is shown in Table 1. The average age was 73.5 years. Every patient had unilateral endophthalmitis (3 left eyes and 3 right eyes). Four patients had DM. Three patients reported visual disturbance as the initial symptom, while the other three initially reported systemic symptoms, such as fatigue, anorexia and algor. The specialties of the initially consulted doctors were as follows: ophthalmologist (3 cases), physician (2 cases), and surgeon (1 case). The diagnosis of EBE caused by GBS was based on positive findings from blood culture testing in 5 of 6 cases. Culture testing of ocular samples was also performed in 3 of these 5 cases, with positive findings in 2 cases. One of 6 cases (case 4) had negative findings in blood culture testing, but had positive findings in ocular sample culture testing, leading us to diagnose EBE caused by GBS. The focus of the infection in the cases was as follows: urinary tract infection (UTI) (3 cases); cellulitis (2 cases); arthritis (1 case); peritonitis (1 case); catheter-associated infection (1 case); and endocarditis (1 case). UTI and cellulitis co-occurred in 2 cases, and arthritis additionally co-occurred in one of these 2 cases. Susceptibility testing used blood samples in all cases except for case 4, for which ocular samples were used (both aqueous and vitreous samples in this case returned the same result). Susceptibility testing revealed that all the strains of GBS in these patients were sensitive to β-lactam antibiotics (penicillins, cephems and carbapenems) and vancomycin. Each strain had varying susceptibility to other antibiotics, such as macrolides (erythromycin and clarithromycin), tetracyclines (minocycline), fluoroquinolone (levofloxacin) and aminoglycoside (arbekacin), although some data were unavailable, as shown in Table 2. Among them, 4 strains were resistant to levofloxacin (no data for 1 isolate). Vitrectomy was performed in 4 cases, including the intravitreal injection of antibiotics. In case 2, vitrectomy could not be performed due to a poor systemic condition. In case 3, the patient’s condition improved with only systemic antibacterial therapy. Visual acuity recovered in 4 cases. In the other cases (cases 4 and 6), visual acuity did not recover even after vitrectomy.

**Table 1:** Clinical details of patients with endogenous endophthalmitis caused by group B streptococcus (present case series)

Case	Age range/Sex	Laterality	Underlying medical condition	Initial symptom	Referring doctor	Number of days between the onset of ocular symptoms and the initial examination by ophthalmologists	Culture testing			Infection focuses	Antibiotics		Surgery	Re-operations	Visual acuity	
							Blood	Ocular sample	Others		Systemic	Intravitreal			Initial	Final (follow up weeks)
1	70-75/[1]	R	DM, uterine cancer	Fever, visual disturbance	Physician	0	(**+**)	Vitreous (**+**)	Urine (**+**)	Peritonitis	IPM/CS	VCM+CAZ	PPV+PEA	(**-**)	0.03	1.0 (35)
2	80-85/[2]	L	Gastric cancer	Fever, visual disturbance	Surgeon	4	(**+**)	N.D	N.D	Catheter-associated infection	CEZ	Not performed	(**-**)	(**-**)	0.03	0.8 (77)
3	70-75/[1]	L	DM, cardiac disease, rheumatoid arthritis	Fever, visual disturbance	Physician	0	(**+**)	N.D	N.D	Endocarditis	IPM/CS	Not performed	(**-**)	(**-**)	CF	1.0 (32)
4	80-85/[1]	L	Uterine cancer	Fatigue, Anorexia	Ophthalmologist	1	(**-**)	Aqueous (**+**), Vitreous (**+**)	Urine (**-**)	UTI	ABPC	VCM+CAZ	PPV	(**-**)	HM	LP (15)
5	60-65/[1]	R	DM	Fatigue, Anorexia	Ophthalmologist	0	(**+**)	Aqueous (**-**), Vitreous (**-**)	Urine (**+**)	UTI, cellulitis, arthritis	ABPC	VCM+CAZ+VRCZ	PPV+IOL removal	PPV for RRD	0.01	0.5 (9)
6	70-75/[1]	R	DM	Algor	Ophthalmologist	1	(**+**)	Aqueous (**+**), Vitreous (**+**)	Urine (**+**)	UTI, cellulitis	ABPC	VCM+CAZ+VRCZ	Anterior chamber irrigation	PPV→ evisceration	LP	Evisceration (1)

Age is described within a 5-year range. Sex is described as [1] or [2]. (+) = positive, (−) = negative or not performed, *N.D* no data, *DM* diabetes mellitus, *UTI* urinary tract infection, *ABPC* ampicillin, *CEZ* cefazolin, *CAZ* ceftazidime, *IPM/CS* imipenem cilastatin, *MEPM* meropenem, *VCM* vancomycin, *VRCZ* voriconazole, *PPV* pars plana vitrectomy, *PEA* phacoemulsification and aspiration, *IOL* intraocular lens, *RRD* rhegmatogenous retinal detachment, *CF* counting fingers, *HM* hand motions, *LP* light perception

**Table 2:** Susceptibility test of antibiotics (Present case series)

Antibiotics/case	1	2	3	4	5	6
Penicillin-G	S	S	S	N/A	S	S
Ampicillin	S	S	S	S	S	S
Cefazolin	N/A	S	S	S	N/A	N/A
Ceftriaxone	S	N/A	S	S	S	S
Meropenem	N/A	S	S	N/A	S	S
Imipenem	S	S	S	S	N/A	N/A
Vancomycin	N/A	S	S	S	S	S
Erythromycin	S	N/A	N/A	N/A	S	S
Clarithromycin	N/A	S	R	N/A	S	N/A
Clindamycin	N/A	S	R	S	S	S
Minocycline	N/A	S	I	R	N/A	S
Arbekacin	N/A	R	R	N/A	NA	NA
Levofloxacin	R	N/A	R	S	R	R

*S* Sensitive, *R* Resistant, *I* Intermediate, *N/A* Not available

A susceptibility test of antibiotics was performed with the microdilution method at each facility. All results were interpreted according to the Clinical and Laboratory Standards Institute standard method.

Susceptibility testing used blood samples in all cases except for case 4, for which ocular samples were used (both aqueous and vitreous samples in this case returned the same result)

### Literature review

**Fig. 1:**
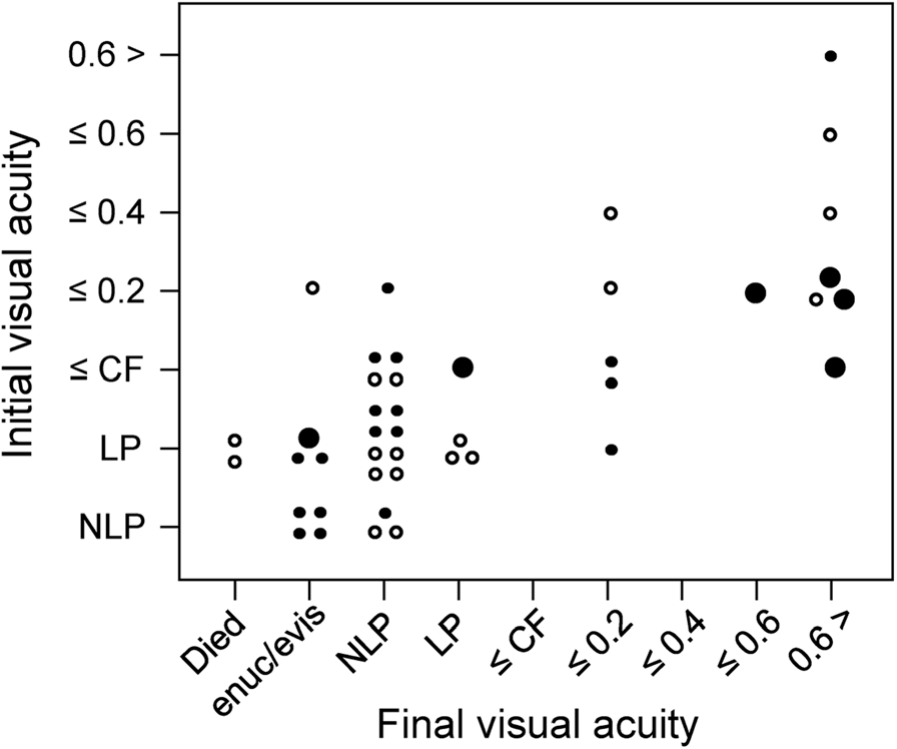
Visual outcome in 43 eyes of 34 patients (from literature review). Visual acuity is shown with a decimal value. Black dots represent data from East Asia and black circles represent data from Western countries. The larger black dots represent data from the cases directly observed by the authors. CF = counting fingers; HM = hand motion; LP = light perception; NLP = no light perception; encu/evis = enucleation/evisceration

We reviewed 41 cases of EBE caused by GBS, including the 6 cases observed at our clinics. Twenty-two cases were from East Asia (16 cases from Japan, 5 cases from Singapore and 1 case from South Korea). The other 19 cases were from Western countries (11 cases from the United States, 5 cases from the United Kingdom, 2 cases from Canada and 1 case from Spain). The 41 cases included 21 men, 18 women and 2 cases with unstated sex. The average age was 65.5 years (SD: 12.7; range: 42–95 years). In 29 patients (71%), EBE was unilateral: 16 cases (55%) in the right eye, 11 (38%) cases in the left eye, and unstated in 2 (7%) cases. Twelve patients (29%) had bilateral EBE. Visual acuity (initial and final) in the 43 eyes of 34 patients for whom these data were available is shown in Fig. 1. Of these 43 eyes, 24 eyes of 19 patients were from East Asia and 19 eyes of 14 patients were from Western countries. Twenty-six eyes (60%) finally lost all vision or died (i.e., no light perception [NLP], phthisis bulbi, evisceration, enucleation or death). Seven eyes (16%) achieved final visual acuities greater than 0.6 in decimal values. There was no significant difference in the visual acuity outcome between the cases from East Asia and Western countries. The group of subjects with a final outcome of loss of vision or death (including NLP, phthisis bulbi, evisceration, enucleation and death) had a significantly greater incidence of initial visual acuity of less than counting fingers (CF) (Table 3). Among the 15 cases that underwent vitrectomy, 10 cases had an initial visual acuity of less than CF. However, 7 of these 10 cases lost vision even after vitrectomy. This rate of vision loss was not significantly different than in the cases with initial visual acuity of less than CF that did not undergo vitrectomy (17/22 cases; 77%). Moreover, a patient age ≥ 80 years at presentation was not associated with a final loss of vision (Table 3). Of 41 patients, 13 (32%) had DM as an underlying medical condition. The second most common condition was human immunodeficiency virus infection (3 cases, 7%). Extraocular infection foci are shown in Table 4. The most common focuses were as follows: endocarditis (15, 37%); arthritis (13, 32%); cellulitis (8, 20%); UTI (6, 15%); pneumonia (4, 10%); and meningitis (3, 7%). These showed no significant differences in the cases from East Asia and Western countries. Of 41 patients, 17 (41%) had infection foci in more than 3 organs (multiple infections in a single type of organ, such as joints or soft tissue, were counted as single foci).

**Table 3:** Visual outcome and clinical background of 43 eyes (Literature review)

Parameters and history	Visual outcome		*P* value
	Loss of vision *N* = 26	More than LP *N* = 17	
Age ≥ 80	6/26 (23%)	3/17 (18%)	NS
Initial visual acuity ≤ CF	24/26 (92%)	8/17 (47%)	0.003

Fisher’s exact test

*LP* light perception, *CF* counting fingers, *NS* not significant

Loss of vision includes non-light perception, phthisis, evisceration, enucleation and death

**Table 4:** Infection focuses of 41 cases (Literature review)

Focuses	East Asia *n* = 22	West *n* = 19	Total *n* = 41
Endocarditis	6 (27%)	9 (47%)	15 (37%)
Arthritis	7 (32%)	6 (32%)	13 (32%)
Cellulitis	6 (27%)	2 (11%)	8 (20%)
Urinary tract infection	3 (14%)	3 (16%)	6 (15%)
Pneumonia	2 (9%)	2 (11%)	4 (10%)
Meningitis	1 (5%)	2 (11%)	3 (7%)
Osteomyelitis	2 (9%)	0	2 (5%)
Periodontitis	2 (9%)	0	2 (5%)
Pharyngitis	0	2 (11%)	2 (5%)
Peritonitis	1 (5%)	0	1 (2%)
Cervical epidural abscess	1 (5%)	0	1 (2%)
Catheter-associated infection	1 (5%)	0	1 (2%)
Diverticulitis	0	1 (5%)	1 (2%)
Endoarteritis	0	1 (5%)	1 (2%)
Unknown	0	1 (5%)	1 (2%)

## Discussion

Our review showed that EBE caused by GBS has an extremely poor prognosis, including vision loss and even death (in 2 cases). Previous reports on invasive GBS infection in nonpregnant adults have also shown a poor prognosis, with a mortality rate as high as 8–24% [6, 34, 35]. Nevertheless, visual acuity recovered in 4 of our 6 directly-observed cases, despite relatively poor initial visual acuity, reaching decimal visual acuity of more than 0.8 in 3 cases. Although a review of 41 cases showed that poor initial visual acuity (less than CF) was significantly associated with a final complete loss of vision, no other clinical characteristics, such as surgical intervention with vitrectomy surgery or age, were predictive of outcome. We also investigated various clinical background factors, such as underlying medical conditions, initial symptoms, the type of referring doctor, the number of days between the onset of ocular symptoms and the initial examination by ophthalmologists, the use of intravitreally injected antibiotics, age, and surgical intervention, but none of these factors were associated with the favourable outcomes we observed. Some reports found differing mortality rates with the differing serotype of the strain of GBS [34, 35]. We did not investigate the serotype of the GBS strains in the current case series, but the differing virulence of each GBS strain might have affected the prognosis, which could explain our finding that some cases had good outcomes despite poor initial visual acuity.

Susceptibility testing of the isolated GBS strains in the 6 directly-observed cases showed that all 6 strains were sensitive to β-lactams (penicillins and cephems) and vancomycin (no data for 1 isolate) and 4 strains were resistant to levofloxacin (no data for 1 isolate). GBS isolates highly resistant to quinolones were first reported in Japan in 2003, followed by reports from other countries [6, 36–39]. While quinolone-resistant GBS is common in Japan (40.2%) and Korea (32.7%), it is rare (0.9 to 4.8%) in other countries [35, 40]. Overuse of quinolone in Japan might have increased the incidence of quinolone-resistant GBS [41]. Thus, intravitreal injection with a combination of vancomycin and ceftazidime, which is commonly used as an empirical treatment before a definite diagnosis, may be a good choice for treating EBE caused by GBS, even before the causative microorganism is identified. As for systemic treatment, although penicillins are usually good choices, the largest case study of adult invasive GBS infection in Japan reported that 9 of 443 (2%) isolate GBS strains had reduced penicillin susceptibility [35], and there has been a report of invasive vancomycin-resistant GBS [42]. Awareness of resistant strains is thus important.

In the current review, the most common non-ocular infection focus was endocarditis (37%) while previous research on invasive GBS infection showed that endocarditis was not a common infection focus [43]. This discrepancy may be due to overestimation caused by selection bias, because endocarditis is severe, and thus more likely to be reported. Nevertheless, the high rate described in the current study is noteworthy, because the mortality rate of endocarditis caused by GBS has been reported to be as high as 40% [44]. The diagnosis of endocarditis requires echocardiography, but the current case series showed significant variation in initial symptoms and the medical field of the first doctor consulted. Thus, it is important for all types of doctors, even ophthalmologists, to consider the possibility of endocarditis on encountering EBE, especially caused by GBS, and to obtain adequate consultation from specialists.

## Conclusions

The past literature has generally reported that EBE caused by GBS is a severe condition with a poor prognosis, especially when initial visual acuity is low, and that loss of vision is common, even after vitrectomy. Nevertheless, 4 of the 6 patients at our clinic had relatively good outcomes, despite low initial visual acuity. Although we cannot confidently speculate why this was so, our results show that good outcomes may still be possible in severe cases, and that every effort should be made to ensure prompt and accurate diagnosis and treatment. Furthermore, our current case series and literature review demonstrates that DM is common in patients with EBE caused by GBS. Finally, the current review highlights that it is important for clinicians to consider the possibility of endocarditis when they encounter EBE, especially when caused by GBS.

## Data Availability

All data are presented in the manuscript, figure, and tables.

## Abbreviations

GBS: Group B streptococcus
EBE: Endogenous bacterial endophthalmitis
DM: Diabetes mellitus
UTI: Urinary tract infection
NLP: No light perception
CF: Counting fingers
